# Is your ice machine really clean? Uncovering the presence of opportunistic pathogens in hospital ice machines

**DOI:** 10.1017/ash.2022.76

**Published:** 2022-05-16

**Authors:** Margot Cazals, Emilie Bedard, Michèle Prévost, Patrice Savard

## Abstract

**Background:** Ice is used in healthcare facilities for medical purposes and consumption by the medical staff and the patients, but some studies have revealed significant microbial contamination of ice machines leading to nosocomial outbreaks or pseudooutbreaks and infections by opportunistic pathogens, including the fungi *Candida*, the bacteria *Pseudomonas aeruginosa*, and nontuberculous mycobacteria (NTM). Although ice machines are complex devices that are prone to contamination, very little is known about their potential as vectors of infections for populations at risk in hospitals. Only few studies document efficient maintenance regimes, specifically cleaning procedures and microbial indicators that would ensure their safe use. **Method:** In this prospective study, combined samples of water and ice, and drain biofilm samples were collected from 36 ice and cold-water distribution machines of a recently built hospital, for a total of 72 samples. Physicochemical parameters (total and free chlorine, temperature, etc) were measured in water, and several opportunistic pathogens (ie, *Candida* spp, *P. aeruginosa*, NTM) and biological indicators (ie, heterotrophic plate counts (HPCs), total and viable bacteria and enterococci) were monitored in water and ice and biofilm. Culture methods were used for HPCs, *Candida* spp, *P. aeruginosa*, and enterococci, and total and viable bacterial populations were estimated using flow cytometry. NTM were monitored by quantitative polymerase chain reaction (qPCR). **Results:** We observed clear differences between the machines in terms of biological contamination, with frequent detection of NTM and presumed *Candida*. Thus, NTM were detected in the 36 samples of ice and water with concentrations from 0.5 to 2×104 gene copies/mL. Among the several species of fungi detected in the ice machines, some were identified as *C. parapsilosis* and *C. guilliermondii*, which are organisms of concern in healthcare facilities. Factors affecting the level of contamination in ice machines include the location of the machines and water quality (ie, temperature and chlorine residual concentration). Depending on the location in the building and the model of ice machine sampled, the biological indicators measurements indicated more or less significant contamination. No link was established between environmental strains recovered from the machines and clinical infections. **Conclusions:** Monitoring results showed that ice machines, while subject to few regulations and controls, can be reservoirs of unsuspected opportunistic pathogens that could lead to nosocomial infections of vulnerable patients. Cleaning procedures should be based on the disinfection of resistant opportunistic pathogens, such as *Candida* and NTM, and the use of general indicators, such as HPCs, should be questioned.

**Funding:** None

**Disclosures:** None

