# Is your ice machine really clean? Uncovering the presence of opportunistic pathogens in hospital ice machines – CORRIGENDUM

**DOI:** 10.1017/ash.2022.273

**Published:** 2022-07-15

**Authors:** Margot Cazals, Emilie Bedard, Michèle Prévost, Patrice Savard

In the above abstract, the author “Michèle Prévost” was originally credited as “Michele Provost”; this error has since been corrected.
